# Nationwide trends in steroid therapy for vestibular neuritis: insights from South Korea’s health insurance review and assessment data

**DOI:** 10.3389/fneur.2025.1560388

**Published:** 2025-04-25

**Authors:** Chul Young Yoon, Tae Hoon Kong, Young Joon Seo, Ji-Yun Park

**Affiliations:** ^1^Research Institute of Hearing Enhancement, Yonsei University Wonju College of Medicine, Wonju, Gangwon-do, Republic of Korea; ^2^Department of Otorhinolaryngology, Yonsei University Wonju College of Medicine, Wonju, Gangwon-do, Republic of Korea; ^3^Department of Neurology, Ulsan University Hospital, University of Ulsan College of Medicine, Ulsan, Republic of Korea

**Keywords:** acute vestibular neuritis, prevalence, big data, health insurance, steroids

## Abstract

**Background:**

While debates persist regarding the benefits and drawbacks of steroid use in treating vestibular neuritis (VN), few studies have analyzed real-world prescription patterns and clinical outcomes. This study aimed to fill this gap by leveraging South Korea’s Health Insurance Review and Assessment (HIRA) big data to explore the actual use of steroids in clinical practice and their associated patient characteristics.

**Methods:**

Using HIRA data from 2007 to 2022, 237,673 VN patients were retrospectively analyzed and categorized into steroid (*n* = 23,235) and non-steroid groups (*n* = 214,438). Demographic, clinical, and economic variables, including age, sex, hospital type, medication use, and costs, were statistically compared using chi-square and t-tests.

**Results:**

Steroid prescriptions accounted for 9.8% of VN cases, predominantly in females (63.2%) and younger patients (2.7% in the 20–24 age group vs. 1.6% in the non-steroid group). Prescription rates declined significantly in patients aged 55 years and older. Outpatients (87.2%) and those treated in clinics (65.1% for males, 75.3% for females) were more likely to receive steroids. Steroid prescriptions were also associated with lower hospital costs and insurance payments compared to the non-steroid group.

**Conclusion:**

This study is the first to analyze real-world steroid usage for VN through big data in Korea, offering valuable insights into clinical practices and prescription trends. Clinicians, especially in primary and outpatient clinic, are more likely to favor steroid treatment and avoid further testing or treatment when they are confident of diagnosing VN. However, the high rate of VN diagnosis in women suggests that vestibular migraine may be underdiagnosed and steroids may be misused. By identifying demographic and economic factors associated with steroid use, the findings highlight the importance of establishing evidence-based guidelines to optimize VN management in clinical settings.

## 1 Introduction

Vestibular neuritis (VN) is an acute vestibular syndrome characterized by the sudden onset of vertigo and dizziness with unilateral vestibular loss, typically persisting for over 24 h (1). The diagnosis of VN relies on a thorough neuro-otologic examination, with key findings including spontaneous horizontal-torsional nystagmus directed toward the unaffected side and a positive head impulse test or caloric paresis on the affected side.

Corticosteroids are frequently prescribed in the acute phase of VN to mitigate inflammation of the vestibular nerve, which is often attributed to viral infection (2). Steroids may alleviate symptoms such as vertigo and dizziness by suppressing immune responses. Administration is typically oral or intravenous, with treatment duration ranging from one to three weeks (3–6). Despite their widespread use, evidence supporting steroid therapy in VN remains limited, as existing studies vary in their protocols regarding initial dosages—commonly between 40 and 60 mg/day—and the duration of administration, which usually spans 7–14 days with a gradual tapering regimen (2, 7–10). Furthermore, some studies have reported no significant long-term benefits of steroid therapy in VN treatment, highlighting the necessity of more comprehensive research (8, 11).

Currently, limited academic research exists on nationwide prescription patterns and outcomes of steroid use in patients with VN. Therefore, a large-scale investigation is needed to assess the role of steroids in treating VN, particularly across diverse clinical settings. South Korea’s Health Insurance Review and Assessment Service (HIRA) provides an ideal resource for such research. The HIRA manages South Korea’s national health insurance system, which covers approximately 98% of the population, and maintains detailed data on patient demographics, diagnoses, prescriptions, and treatments (12).

This study aimed to use the extensive healthcare data of HIRA to evaluate current clinical practices concerning steroid therapy for VN in South Korea. By analyzing data on prescription patterns, patient outcomes, and regional variations in treatment, this study also aimed to identify the differences in sex, hospital type, and concomitant medication use in the context of steroid administration, providing evidence-based insights into the efficacy of steroids in treating VN. The ultimate objective was to establish treatment guidelines tailored to the domestic clinical environment to ensure that patients receive appropriate care.

## 2 Materials and methods

### 2.1 Data collection and study population

**Figure 1:**
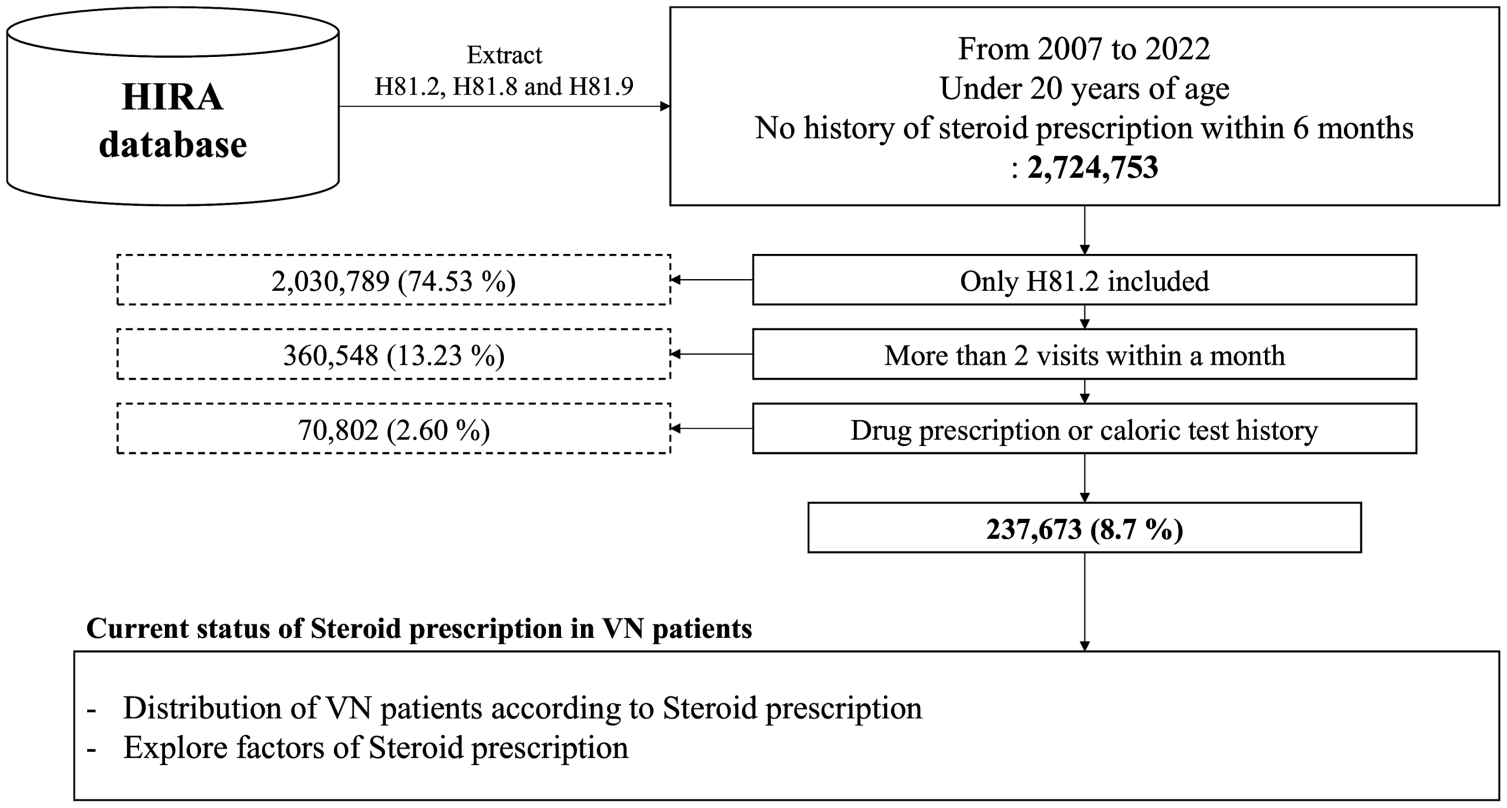
Flow diagram of study and participant selection.

The study design was approved by the Institutional Review Board of Ulsan University Hospital (UUH 2023-07-030). This study used data from the HIRA. The study participants were included if they had VN codes in their billing records between 2007 and 2022. Individuals under 20 years of age or with a history of steroid prescriptions within 6 months of the diagnosis date were excluded. A total of 2,724,753 patients were included based on these criteria, with additional selection through operational definitions (Figure 1) (13).

Three ICD-10 codes were included; however, only the code for VN, H81.2, was used (Supplementary tables in Supplementary information). Patients who visited the hospital only once a month were excluded, as they could have been misdiagnosed or had unclear symptoms. Therefore, only patients who visited the hospital two or more times within a month were included. The prescription of five types of drugs (antiemetics, benzodiazepines, antihistamines, steroids, and antiplatelets) and the presence of a caloric test were included in the criteria, as these factors were expected to influence the diagnostic specificity for VN (Supplementary tables in Supplementary information). Patients were considered to have VN if they had a history of drug prescription or caloric testing. Ultimately, the study included 237,673 participants. Demographics provided patient counts, percentages, and *p*-values indicating statistical significance, which analyze how each variable is distributed across the patient population.

### 2.2 Study design

This study (1) assessed the current status of the diagnosis, treatment, and management of VN in South Korea and (2) elucidated the prescription status of steroids (Figure 1). For (1), the distribution of the patients was examined based on sex, age, drug/treatment, inpatient or outpatient status, cost, and hospital type. For (2), an analysis was conducted based on sex, age, drug/treatment, inpatient/outpatient status, cost, and hospital type according to steroid prescriptions.

Age was analyzed in two ways: categorized into 5-year intervals (nominal age) and maintained as a continuous variable (continuous age) with 1-year increments. Drug analysis excluded steroids used (2) and focused on antiemetics, benzodiazepines, antihistamines, and antiplatelet agents. Treatment analyses were performed for the caloric tests. Whether there are more VN patients among females than males was unclear; therefore, the analysis was divided into male/female, inpatients/outpatients, and hospital types, and how this phenomenon affects steroid prescriptions was evaluated. All variables related to monetary amounts were converted into US dollars.

### 2.3 Statistical analysis

Categorical variables were analyzed using the chi-square test, whereas continuous variables were analyzed using t-tests. Percentages were reported for categorical variables, and confidence intervals (95%) were reported for continuous variables. All statistical analyses were performed using SAS, version 9.4 (SAS Institute Inc., Cary, NC, USA).

## 3 Results

### 3.1 Demographics of patients with VN

Between 2007 and 2022, 237,673 patients were categorized according to variables such as sex, age group, medication usage, treatment methods, patient type, and hospital type (Table 1). More patients were women than men (65.6%), with a women-to-men ratio of 1.9:1 (*p* < 0.0001). VN was the most prevalent in the sixth decade, representing 12.4% of all patients.

**Table 1:** Statistical analysis of factors related to vestibular neuritis diagnosis and steroid prescription patterns in patients.

Variable		Number of patients, %	Steroid group, %	Non-steroid group, %
All^*^		237,673	23,235 (9.8)	214,438 (90.2)
Sex^*, **^	Male	81,737 (34.4)	8,559 (36.8)	73,178 (34.1)
	Female	155,936 (65.6)	14,676 (63.2)	141,260 (65.9)
Nominal age^*, **^	20–24	4,074 (1.7)	619 (2.7)	3,455 (1.6)
	25–29	6,572 (2.8)	994 (4.3)	5,578 (2.6)
	30–34	9,861 (4.2)	1,393 (6)	8,468 (4)
	35–39	14,250 (6)	1,896 (8.2)	12,354 (5.8)
	40–44	17,942 (7.6)	2,186 (9.4)	15,756 (7.4)
	45–49	22,753 (9.6)	2,524 (10.9)	20,229 (9.4)
	50–54	27,673 (11.6)	2,887 (12.4)	24,786 (11.6)
	55–59	29,508 (12.4)	2,874 (12.4)	26,634 (12.4)
	60–64	28,189 (11.9)	2,498 (10.8)	25,691 (12)
	65–69	26,397 (11.1)	2,054 (8.8)	24,343 (11.4)
	70–74	22,455 (9.5)	1,549 (6.7)	20,906 (9.8)
	75–79	16,653 (7)	1,053 (4.5)	15,600 (7.3)
	80–84	8,217 (3.5)	525 (2.3)	7,692 (3.6)
	85–89	2,620 (1.1)	159 (0.7)	2,461 (1.2)
	90–94	465 (0.2)	19 (0.1)	446 (0.2)
	95+	44 (0)	5 (0)	39 (0)
Drug	Antiemetic^*, **^	14,128 (5.9)	1,281 (5.5)	12,847 (6)
	Benzodiazepines^*, **^	81,667 (34.4)	4,504 (19.4)	77,163 (36)
	Antihistamine^*, **^	165,154 (69.5)	11,289 (48.6)	153,865 (71.8)
	Antiplatelet^*, **^	9,699 (4.1)	287 (1.2)	9,412 (4.4)
	Steroid^*^	23,235 (9.8)	-	-
Diagnostic test	Caloric test^*, **^	20,824 (8.8)	937 (4)	19,887 (9.3)
Patient’s type^*, **^	Inpatients	44,540 (18.7)	3,346 (12.8)	41,194 (17.4)
	Outpatients	197,486 (83.1)	20,251 (87.2)	177,235 (82.7)
Hospital type (male)^*, **^	Tertiary	7,505 (3.7)	649 (7.6)	6,856 (9.4)
	General hospital	20,052 (8.4)	1,705 (19.9)	18,347 (25.1)
	Hospital	4,705 (2)	518 (6.1)	4,187 (5.7)
	Clinic	48,011 (20.2)	5,570 (65.1)	42,441 (58)
	Pharmacy	1,464 (0.6)	117 (1.4)	1,347 (1.8)
Hospital type (female)^*, **^	Tertiary	9,212 (3.9)	692 (4.7)	8,520 (6)
	General hospital	28,198 (11.9)	1,940 (13.2)	26,258 (18.6)
	Hospital	8,247 (3.5)	750 (5.1)	7,497 (5.3)
	Clinic	107,441 (45.2)	11,052 (75.3)	96,389 (68.2)
	Pharmacy	2,838 (1.2)	242 (1.7)	2,596 (1.8)

Variable		Mean (CI)	Steroid group, CI	Non-steroid group, CI
Continuous variable^*, **^	Age	54.8 (54.6, 54.9)	52.7 (52.5, 52.9)	56.9 (56.8, 56.9)
	Hospital costs ($)	64.7 (63.7, 65.8)	62.8 (61.2, 64.4)	66.7 (66.2, 67.1)
	Cost of patient-paid ($)	19.7 (19.4, 20.1)	19.1 (18.7, 19.6)	20.4 (20.2, 20.5)
	Cost of insurance-paid ($)	44.9 (44.2, 45.7)	43.6 (42.5, 44.8)	46.2 (45.9, 46.6)

^*^The distribution of VN patients differs statistically significantly (*p* < 0.05) according to this variable.

^**^This variable shows a statistically significant difference (*p* < 0.05) based on the presence or absence of steroid prescriptions.

CI, confidence intervals.

Antihistamines were the most commonly prescribed medications, with 69.5% of patients receiving them. Benzodiazepines were prescribed to 34.4% of patients, and antiemetics was prescribed to 5.9% of patients. Steroids were prescribed to 9.8% of patients. All medications showed significant differences in usage (*p* < 0.0001). Caloric testing was administered to 8.8% of patients, highlighting its importance in diagnosis and treatment. Additionally, 83.1% of patients were treated as outpatients, whereas 18.7% were inpatients. The predominance of outpatient treatment was significant (*p* < 0.0001).

Both male and female patients predominantly received treatment at clinics. Among males, 20.2% were treated at clinics, whereas 45.2% of females received clinic-based treatment. Fewer patients were treated at general or tertiary hospitals; however, differences in hospital type between the sexes were also significant (*p* < 0.0001). In terms of continuous variables, the mean patient age was 54.8 years. The average hospital cost was $64.7, with patients paying an average of $19.7 and insurance covering an average of $44.9. These continuous variables also showed significant differences (*p* < 0.0001).

**Figure 2:**
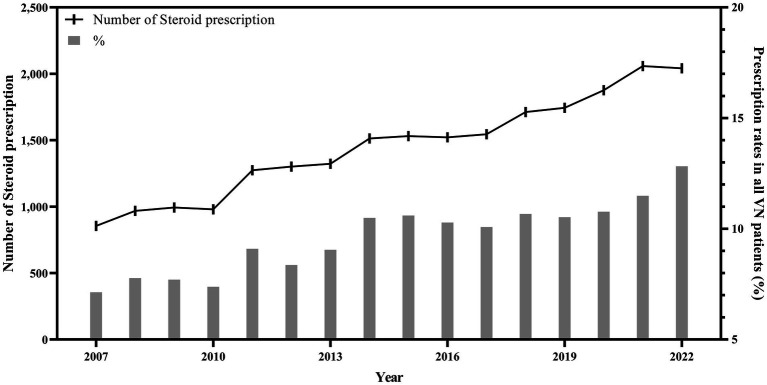
Steroid group per whole patient according to each variable.

Figure 2 shows the number of steroid prescriptions and the proportion of patients with VN who received steroids from 2007 to 2022. The number of prescriptions increased approximately threefold, from approximately 400 in 2007 to approximately 1,200 in 2022. Additionally, the proportion of patients with VN who were prescribed steroids increased from approximately 7–13% during this period.

### 3.2 Current status of steroid prescription

A significant difference was observed in the number of patients prescribed steroids among the total number of patients, with significant differences observed for all variables. More prescriptions were observed among females than among males, even in the steroid prescription group. In terms of nominal and continuous age, steroid prescriptions were higher in the relatively younger patient groups than in older patients. Regarding drugs and treatment, the proportion of targeted patients was lower in the steroid prescription group than in the non-steroid prescription group. However, a smaller decrease was observed in the proportion of antiemetics than in the other variables. The proportion of outpatients was higher in the steroid prescription group than in the non-steroid prescription group, and the proportion of patients receiving steroid prescriptions was higher in clinics than in other hospital types, regardless of sex. Hospital costs, costs of patient-paid care, and costs of insurance paid were lower in the steroid prescription group than in the non-steroid prescription group.

**Figure 3:**
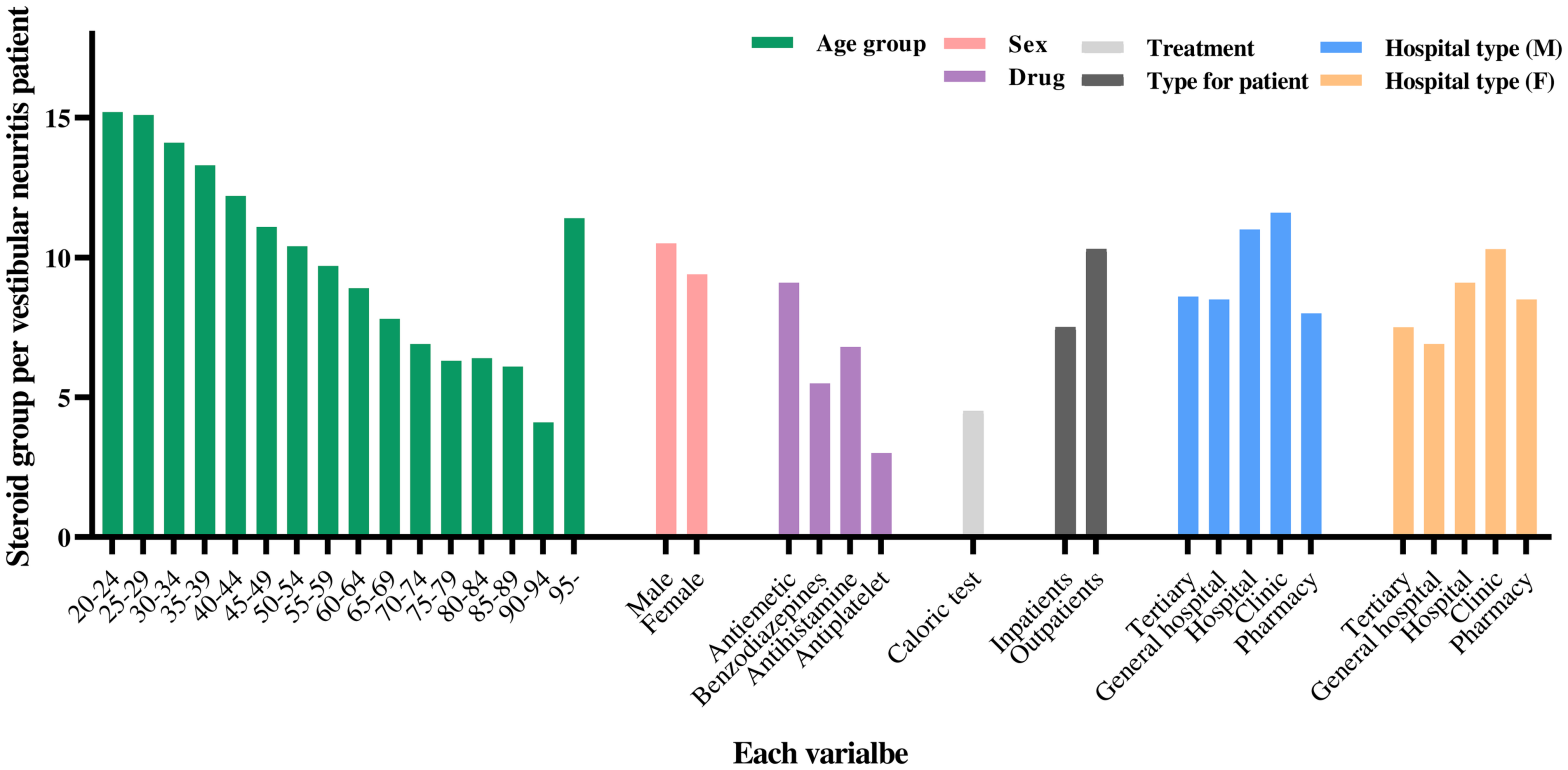
Steroid prescription according to year, from 2007 to 2022.

A total of 23,235 patients (9.8%) were prescribed steroids, whereas 214,438 (90.2%) were not (Table 1). This difference was significant (*p* < 0.0001), with variations observed across sex, age, medication use, treatment method, and hospital type. A total of 8,559 males (36.8%) and 14,676 females (63.2%) received steroids, showing a significant difference (*p* < 0.0001). The differences across age groups were also significant (*p* < 0.0001). Younger patients were more likely to be prescribed steroids, with 2.7% of patients in the steroid group aged 20–24 compared to 1.6% in the non-steroid group. However, the prescription rate decreased among patients aged 55 years and older, with the non-steroid group having a higher proportion of prescriptions than the steroid group in patients aged 55–59 and older. Over 15% of the patients with VN in their 20s were prescribed steroids, but less than 7% of those in their 70s and older were prescribed steroids (Figure 3).

The use of other medications was less frequent among patients who were prescribed steroids. Antihistamines were the most commonly prescribed drugs among steroid users, with 48.6% of steroid-prescribed patients receiving steroids compared to 71.8% of patients not receiving steroids. Differences were also observed in the use of benzodiazepines and antiemetics, with significant variations in all medication categories. Caloric testing was performed on 4.0% of steroid-prescribed patients compared to 9.3% of non-steroid-prescribed patients. Additionally, 87.2% of the steroid users were outpatients, whereas only 12.8% were inpatients, indicating that outpatients were significantly more likely to receive steroids (*p* < 0.0001).

Both male and female patients were most likely to be prescribed steroids in clinics, with relatively fewer prescriptions in tertiary or general hospitals. Additionally, the average age of steroid-prescribed patients was 52.7 years, compared to 56.9 years for non-prescribed patients, indicating that younger patients were more likely to receive steroids. Hospital costs were also lower for patients prescribed steroids, with similar trends observed for both patient- and insurance-paid costs. Due to the uncertainty in the VN diagnosis, the group that was relatively prescribed steroids could be considered as the group with a more definitive diagnosis. It is possible that, by quickly identifying evidence for VN and actively prescribing steroids, the number of hospital visits was reduced, leading to lower costs.

In summary, this Table 1 highlights the significant differences in patient characteristics based on steroid prescription status. Significant variations were observed in sex, age, medication use, and hospital type, suggesting that steroids were more frequently prescribed to certain patient groups.

## 4 Discussion

This longitudinal study analyzed the clinical treatment patterns for VN using health insurance claims data from South Korea. The primary findings can be summarized as follows: (1) Steroid prescription rates for VN in South Korea were low at 9.8% for the entire period, with cases increasing threefold from 400 in 2007 to 1,200 in 2022. Our opinion is that the sample size is too large due to the overuse of the VN code in clinical settings. Continuous education and awareness improvement in South Korea have led to increased steroid treatment for VN patients. As a result, steroid treatment has steadily increased within South Korea. Steroids were predominantly prescribed in primary care clinics, where 65.4% of diagnoses occurred. In outpatient setting, physicians are likely to prescribe steroids more frequently because they prefer a quicker and more aggressive way to improve symptoms compared with inpatients, who can be monitored over a longer period of time. (2) Steroids were more frequently prescribed to younger patients because of a lower risk of side effects. In contrast, the prescription rates for women are higher than for men, especially in primary and secondary care (with a 2:1 female to male ratio). Many cases of vestibular neuritis rather than vestibular migraine in women may in fact be overdiagnosed. (3) The average hospital cost per patient was $64.7, with 69.4% covered by insurance; costs were lower for the steroid prescription group, likely because these prescriptions were primarily managed in cost-effective primary care settings.

Current evidence offers a nuanced perspective on corticosteroid therapy for VN. Several randomized controlled trials (RCTs) have investigated the efficacy of corticosteroids in VN treatment. A meta-analysis of four RCTs involving 182 patients demonstrated that corticosteroid therapy significantly improved objective measures, such as canal paresis, compared with vestibular rehabilitation therapy after one month (14). Another systematic review and meta-analysis supported the use of corticosteroids for achieving complete caloric recovery and reducing canal paresis in patients with VN (4). However, a Cochrane review of four RCTs with 149 patients found insufficient evidence to recommend corticosteroid use for idiopathic acute vestibular dysfunction, citing methodological biases and inconsistent treatment protocols (15). Although the efficacy of corticosteroids in VN remains controversial, their prescriptions have increased approximately threefold. In addition, the potential adverse effects, such as hyperglycemia or the risk of infection, highlight the need for careful patient selection and monitoring during corticosteroid therapy (16). Recent literature has further emphasized the evolving understanding of VN diagnosis and the practical use of corticosteroids, particularly in emergency and primary care settings (17, 18).

Establishing appropriate guidelines is critical to improving the effectiveness and reducing the misuse of steroid therapy for acute VN. This is especially important in primary care clinics, where 65.4% of patients are diagnosed, and steroid prescription rates remain high. Practical and evidence-based guidelines are needed to standardize treatment practices in these settings. This study identified significant differences in sex, age, hospital type, medication, and hospital costs associated with steroid use. The increased use of steroids in younger people is likely owing to the safety of their side effects. Additionally, younger patients may present earlier and be perceived to have better prognoses based on age and fitness, which could introduce a bias toward prescribing steroids. However, the higher prescription of steroids in women may be owing to inappropriate prescriptions for vertigo, such as vestibular migraine, which is more common in women (19, 20). Notably, the overall frequency of VN diagnoses was similar in men and women.

The observation that female patients accounted for approximately twice the number of VN diagnoses in primary and secondary hospitals, with over 45% diagnosed at primary clinics, raises important considerations for researchers, including the potential influence of gender-specific factors such as hormonal differences, healthcare-seeking behavior, or diagnostic biases. Additionally, the high proportion of diagnoses at primary clinics suggests the need to evaluate accessibility, diagnostic accuracy, and treatment protocols in these settings to ensure optimal care outcomes. The difference in caloric test utilization suggests that clinicians may be uncertain about the diagnosis of vestibular neuritis and may have a selection bias when ordering the test. If the clinician perceives the diagnosis as straightforward, steroids are more likely to be prescribed, and the caloric test may be considered unnecessary. However, if there is diagnostic uncertainty, the clinician may be more inclined to prescribe steroids (to cover the possibility of vestibular neuritis) and order the caloric test to confirm their clinical suspicion. Health insurance data in Korea is a massive dataset that covers 98% of the entire population. Our study included all patients with a VN diagnosis code, so we could not verify the diagnostic procedures of individual hospitals or physicians. Therefore, we cannot be certain that the VN code was assigned based on an accurate diagnosis, and we particularly observed that the diagnosis of VN in women was excessively prevalent in clinical settings. Of the average hospital cost of $64.7 per patient, $44.9 (69.4%) was covered by insurance, reflecting effective management within South Korea’s National Health Insurance system (21). The analysis further revealed that patients in the steroid prescription group incurred lower hospital costs, likely owing to the cost-effectiveness of treatment at clinics.

While the use of steroids in VN has been a topic of ongoing debate, with arguments both for and against their efficacy, there has been a lack of research analyzing how steroids are actually prescribed and utilized in real-world clinical settings. This study is significant as it provides the first comprehensive analysis of steroid use for VN based on real-world data from South Korea’s national healthcare database. By leveraging big data, this research offers valuable insights into prescription patterns, patient characteristics, and associated clinical outcomes, filling a critical gap in the literature. These findings not only reflect current practices but also serve as a foundation for developing evidence-based guidelines tailored to real-world clinical environments.

We additionally addressed the potential overuse of VN diagnostic codes and the underutilization of caloric testing. Due to the lack of standardized diagnostic confirmation, especially in the outpatient setting, VN may have been over diagnosed based on broad clinical impressions. At the same time, the low rate of caloric test utilization suggests limited objective verification, likely leading to the inclusion of patients without definite VN. These factors may have contributed to an overestimation of VN cases in the dataset and should be considered when interpreting the results.

This study has some limitations. Other vestibular function tests, like the video head impulse test (vHIT), were not included in the study because they were not covered by insurance during the study period. vHIT has been covered by insurance in Korea only since 2021, which is the midpoint of our study period. Therefore, we were inevitably unable to include vHIT in our analysis. Additionally, because of the nature of big data analysis, treatment guidelines cannot be directly derived, as diagnoses were determined using administrative codes without consideration of vestibular test results or clinical courses.

In conclusion, steroid treatment for VN is increasing and is the most commonly prescribed treatment in clinics, with a higher proportion of prescriptions in women and younger people. A comprehensive analysis of steroid use and associated factors is essential to optimize VN treatment. Establishing accurate and evidence-based guidelines can reduce inappropriate steroid and medication use, improve treatment outcomes, and minimize unnecessary healthcare costs. This study also fills a significant gap in the academic literature and potentially establishes future clinical guidelines for the management of VN, both in South Korea and internationally. However, given the heterogeneity and limitations of existing studies, further robust randomized controlled trials are essential to provide definitive guidance on the role of corticosteroids in VN management.

## Data Availability

The original contributions presented in the study are included in the article/Supplementary material, further inquiries can be directed to the corresponding author.
