# Supplementary material for: Nationwide trends in steroid therapy for vestibular neuritis: insights from South Korea’s health insurance review and assessment data
Source: Front Neurol. 2025 Apr 25;16:1560388. doi: 10.3389/fneur.2025.1560388 (PMC12061983; doi:10.3389/fneur.2025.1560388)
Supplement: Supplementary file 1 [file Table_1.DOCX]

Supplementary Material

# Supplementary Table

**Supplement 1-1. Used factors for operational definition**

| ICD-10 | | Diagnosis | | | | | |
| --- | --- | --- | --- | --- | --- | --- | --- |
| H81.2 | | Vestibular neuronitis | | | | | |
| H81.8 | | Other disorders of vestibular function | | | | | |
| H81.9 | | Disorder of vestibular function, unspecified | | | | | |
| Type of drug | **Ingredient code** | | | | | | |
| Antiemetic | 483001ACH | | 167303BIJ | 204605BIJ | 193701ACH | 193707CSI | 367400ATB |
|  | 483002ACH | | 204601ATB | 490001BIJ | 193702BIJ | 193709ALQ | 193730BIJ |
|  | 616001BIJ | | 204603ATB | 490002BIJ | 193703BIJ | 294900ATB | 193708ATR |
|  | 167301ATB | | 204606ASY | 222601ATD | 193704APD | 193801ATR | 461700ATB |
|  | 167301ATD | | 204602BIJ | 222501BIJ | 193705ALQ | 193710ALQ | 461700ATE |
|  | 167301BIJ | | 204604BIJ | 193701ATB | 193706CSI | 295500ATB |  |
| Benzodiazepines | 160601ATB | | 243501ATB | 105505ATR | 137102ACH | 156202ATB | 194201ATB |
|  | 161801ATB | | 243502ATB | 118501ATB | 137302ATB | 156501ATB | 250501ATB |
|  | 195201BIJ | | 105501ATB | 131201ATB | 142901ATB | 156502ATB | 250702ATR |
|  | 195202BIJ | | 105502ATB | 131202ATB | 142902ATB | 156503ATB | 250703ATR |
|  | 195203BIJ | | 105504ATB | 135702ATB | 142903ATB | 185501ATB | 250704ATD |
|  | 195204BIJ | | 105505ATB | 137101ACH | 156201ATB | 185504ATB |  |
| Antihistamine | 453902ATB | | 131801ATB | 145901ATB | 151302ACR | 185401ACS | 355901ATB |
|  | 521601ATB | | 131802BIJ | 150601ATB | 152501ATB | 185401ATB | 206201ATB |
|  | 130501ATB | | 260000ASY | 150602ALQ | 463601ATB | 185402ASY | 213301ATB |
|  | 259900ACH | | 135201ATB | 260100ACR | 463602ALQ | 190501ASY | 213301BIJ |
|  | 259900ATB | | 551501ATB | 151301ACR | 524800ATB | 190502ATB |  |
| Steroids | 116401ATB | | 141903ATB | 170901ATB | 193305ATB | 193603BIJ | 217302BIJ |
|  | 116502BIJ | | 141904ATB | 170906ATB | 193501BIJ | 193604BIJ |  |
|  | 296900ATB | | 142201BIJ | 171201BIJ | 193502BIJ | 217001ATB |  |
|  | 140801ATB | | 142202BIJ | 171202BIJ | 193601BIJ | 217003ASY |  |
|  | 141901ATB | | 160201ATB | 193302ATB | 193602BIJ | 217004ASY |  |
| Antiplatelet | 244101ACE | | 133201ATB | 111001ATB | 110704ATB | A61800ATE | 110901BIJ |
|  | 244101ACH | | 133201ATD | 111001ATE | 110705ACE | 394500ATB | 110902BIJ |
|  | 244102ACH | | 133201ATR | 111002ATE | 110706ATB | 489700ACR | B21200ATB |
|  | 136901ATB | | 133202APD | 111003ACE | 252900ATB | 517900ACE | 259000ATB |
|  | 136902ATB | | 133202ATB | 111003ATE | 253000ATB | 517900ACH | 259100ACH |
|  | 492501ATB | | 133202ATD | 111001ACH | 254200ATB | 517900ATE | A20100ATB |
|  | 495201ATB | | 133204ATR | 111003ATB | 256800ATB | 582600ATE |  |
|  | 498801ATB | | 506100ATB | 110701ATB | 263300ACH | 667500ACE |  |
|  | 501501ATB | | 687200ATR | 110701ATE | A05500ATB | 110801ATB |  |
|  | 133203ATR | | 133203ACR | 110702ATB | A05500ATE | 110802ATB |  |
|  | 133201ACR | | 111001ACE | 110703ATE | A34400ATB | D87600ATE |  |
| Type of test | **Test code** | | | | | | |
| Caloric | F6928 | | F6324 | F6334 | F6335 | F6893 | F6894 |

**Supplement 1-2. Detail of used drug**

| **Type of drug** | **Ingredient code** | | | | **Ingredient information** |
| --- | --- | --- | --- | --- | --- |
| Antiemetic | 483001ACH | | | | aprepitant 80mg |
|  | 483002ACH | | | | aprepitant 125mg |
|  | 616001BIJ | | | | fosaprepitant dimeglumine (as fosaprepitant) 157.5mg |
|  | 167301ATB | | | | granisetron (HCl) 1mg |
|  | 167301ATD | | | | granisetron (HCl) 1mg |
|  | 167301BIJ | | | | granisetron (HCl) 1mg |
|  | 167303BIJ | | | | granisetron (HCl) 3mg |
|  | 204601ATB | | | | ondansetronhydrochloridedihydrate (asondansetron) 4mg |
|  | 204603ATB | | | | ondansetronhydrochloridedihydrate (asondansetron) 8mg |
|  | 204606ASY | | | | ondansetronhydrochloridehydrate (asondansetron) 12mg |
|  | 204602BIJ | | | | ondansetronhydrochloridedihydrate (asondansetron) 24mg |
|  | 204604BIJ | | | | ondansetronhydrochloridedihydrate (asondansetron) 4mg |
|  | 204605BIJ | | | | ondansetronhydrochloridedihydrate (asondansetron) 8mg |
|  | 490001BIJ | | | | palonosetron HCl (asPalonosetron0.25mg) 0.28mg |
|  | 490002BIJ | | | | palonosetron HCl (asPalonosetron0.075mg) 0.084mg |
|  | 222601ATD | | | | ramosetron HCl 0.1mg |
|  | 222501BIJ | | | | ramosetron 0.3mg |
|  | 193701ATB | | | | metoclopramide |
|  | 193701ACH | | | | metoclopramide HCl |
|  | 193702BIJ | | | | metoclopramide HCl |
|  | 193703BIJ | | | | metoclopramide HCl |
|  | 193704APD | | | | metoclopramide HCl |
|  | 193705ALQ | | | | metoclopramide HCl |
|  | 193706CSI | | | | metoclopramide HCl |
|  | 193707CSI | | | | metoclopramide HCl |
|  | 193709ALQ | | | | metoclopramide HCl |
|  | 294900ATB | | | | metoclopramide HCl |
|  | 193801ATR | | | | metoclopramide HCl |
|  | 193710ALQ | | | | metoclopramide HCl |
|  | 295500ATB | | | | metoclopramide HCl |
|  | 367400ATB | | | | metoclopramide HCl |
|  | 193730BIJ | | | | metoclopramide hydrochloride |
|  | 193708ATR | | | | metoclopramide hydrochloride hydrate (as metoclopramide hydrochloride) |
|  | 461700ATB | | | | metoclopramide hydrochloride (as metoclopramide) |
|  | 461700ATE | | | | metoclopramide hydrochloride (as metoclopramide 5mg) |
| Benzodiazepines | | | 160601ATB | | flunitrazepam 1mg |
|  |  |  | 161801ATB | | flurazepam HCl 15mg |
|  |  |  | 195201BIJ | | midazolam 15mg |
|  |  |  | 195202BIJ | | midazolam 5mg |
|  |  |  | 195203BIJ | | midazolam 50mg |
|  |  |  | 195204BIJ | | midazolam 3mg |
|  |  |  | 243501ATB | | triazolam 125㎍ |
|  |  |  | 243502ATB | | triazolam 250㎍ |
|  |  |  | 105501ATB | | alprazolam 1mg |
|  |  |  | 105502ATB | | alprazolam 250㎍ |
|  |  |  | 105504ATB | | alprazolam 400㎍ |
|  |  |  | 105505ATB | | alprazolam 500㎍ |
|  |  |  | 105505ATR | | alprazolam 500㎍ |
|  |  |  | 118501ATB | | bromazepam 3mg |
|  |  |  | 131201ATB | | chlordiazepoxide HCl 10mg |
|  |  |  | 131202ATB | | chlordiazepoxide HCl 5mg |
|  |  |  | 135702ATB | | clobazam 5mg |
|  |  |  | 137101ACH | | clorazepatedipotassium 10mg |
|  |  |  | 137102ACH | | clorazepatedipotassium 5mg |
|  |  |  | 137302ATB | | clotiazepam 5mg |
|  |  |  | 142901ATB | | diazepam 10mg |
|  |  |  | 142902ATB | | diazepam 2mg |
|  |  |  | 142903ATB | | diazepam 5mg |
|  |  |  | 156201ATB | | ethylloflazepate 1mg |
|  |  |  | 156202ATB | | ethylloflazepate 2mg |
|  |  |  | 156501ATB | | etizolam 1mg |
|  |  |  | 156502ATB | | etizolam 500㎍ |
|  |  |  | 156503ATB | | etizolam 250㎍ |
|  |  |  | 185501ATB | | lorazepam 1mg |
|  |  |  | 185504ATB | | lorazepam 500㎍ |
|  |  |  | 194201ATB | | mexazolam 0.5mg |
|  |  |  | 250501ATB | | zolpidem 10mg |
|  |  |  | 250702ATR | | zolpidemtartrate 6.25mg |
|  |  |  | 250703ATR | | zolpidemtartrate 12.5mg |
|  |  |  | 250704ATD | | zolpidemtartarate 10mg |
| Antihistamine | | 453902ATB | | bepotastinebesilate 10mg | |
|  |  | 521601ATB | | bepotastinesalicylate 9.64mg | |
|  |  | 130501ATB | | cetirizine HCl 10mg | |
|  |  | 259900ACH | | cetirizine HCl 5mg | |
|  |  | 259900ATB | | cetirizine HCl 5mg | |
|  |  | 131801ATB | | chlorpheniraminemaleate 2mg | |
|  |  | 131802BIJ | | chlorpheniraminemaleate 4mg | |
|  |  | 260000ASY | | chlorpheniraminemaleate 0.4mg | |
|  |  | 135201ATB | | clemastinefumarate 1.34mg | |
|  |  | 551501ATB | | desloratadine 5mg | |
|  |  | 145901ATB | | dimenhydrinate 50mg | |
|  |  | 150601ATB | | ebastine 10mg | |
|  |  | 150602ALQ | | ebastine 1mg | |
|  |  | 260100ACR | | ebastine 10mg | |
|  |  | 151301ACR | | emedastinefumarate 1mg | |
|  |  | 151302ACR | | emedastinefumarate 2mg | |
|  |  | 152501ATB | | epinastine HCl 10mg | |
|  |  | 463601ATB | | levocetirizine 5mg | |
|  |  | 463602ALQ | | levocetirizine HCl 0.5mg | |
|  |  | 524800ATB | | levocetirizine 2.5mg | |
|  |  | 185401ACS | | loratadine 10mg | |
|  |  | 185401ATB | | loratadine 10mg | |
|  |  | 185402ASY | | loratadine 1mg | |
|  |  | 190501ASY | | mequitazine 500㎍ | |
|  |  | 190502ATB | | mequitazine 5mg | |
|  |  | 355901ATB | | mizolastine 10mg | |
|  |  | 206201ATB | | oxatomide 30mg | |
|  |  | 213301ATB | | piprinhydrinate 3mg | |
|  |  | 213301BIJ | | piprinhydrinate 3mg | |
| Antiplatelet | | 244101ACE | | triflusal | |
|  |  | 244101ACH | | triflusal | |
|  |  | 244102ACH | | triflusal | |
|  |  | 136901ATB | | clopidogrel bisulfate (as clopidogrel) | |
|  |  | 136902ATB | | clopidogrel bisulfate (as clopidogrel) | |
|  |  | 492501ATB | | clopidogrel resinate (as clopidogrel) | |
|  |  | 495201ATB | | clopidogrel besylate (as clopidogrel) | |
|  |  | 498801ATB | | clopidogrel napadisilate monohydrate (as clopidogrel) | |
|  |  | 501501ATB | | clopidogrel camsylate (as clopidogrel) | |
|  |  | 133203ATR | | cilostazol | |
|  |  | 133201ACR | | cilostazol | |
|  |  | 133201ATB | | cilostazol | |
|  |  | 133201ATD | | cilostazol | |
|  |  | 133201ATR | | cilostazol | |
|  |  | 133202APD | | cilostazol | |
|  |  | 133202ATB | | cilostazol | |
|  |  | 133202ATD | | cilostazol | |
|  |  | 133204ATR | | cilostazol | |
|  |  | 506100ATB | | cilostazol | |
|  |  | 687200ATR | | cilostazol | |
|  |  | 133203ACR | | cilostazol (micronized) | |
|  |  | 111001ACE | | aspirin (enteric coated) (as aspirin) | |
|  |  | 111001ATB | | aspirin (enteric coated) (as aspirin) | |
|  |  | 111001ATE | | aspirin (enteric coated) (as aspirin) | |
|  |  | 111002ATE | | aspirin (enteric coated) (as aspirin) | |
|  |  | 111003ACE | | aspirin (enteric coated) (as aspirin) | |
|  |  | 111003ATE | | aspirin (enteric coated) (as aspirin) | |
|  |  | 111001ACH | | aspirin (enteric coated) | |
|  |  | 111003ATB | | aspirin (enteric coated) | |
|  |  | 110701ATB | | aspirin | |
|  |  | 110701ATE | | aspirin | |
|  |  | 110702ATB | | aspirin | |
|  |  | 110703ATE | | aspirin | |
|  |  | 110704ATB | | aspirin | |
|  |  | 110705ACE | | aspirin | |
|  |  | 110706ATB | | aspirin | |
|  |  | 252900ATB | | aspirin | |
|  |  | 253000ATB | | aspirin | |
|  |  | 254200ATB | | aspirin | |
|  |  | 256800ATB | | aspirin | |
|  |  | 263300ACH | | aspirin | |
|  |  | A05500ATB | | aspirin | |
|  |  | A05500ATE | | aspirin | |
|  |  | A34400ATB | | aspirin | |
|  |  | A61800ATE | | aspirin | |
|  |  | 394500ATB | | aspirin | |
|  |  | 489700ACR | | aspirin | |
|  |  | 517900ACE | | aspirin | |
|  |  | 517900ACH | | aspirin | |
|  |  | 517900ATE | | aspirin | |
|  |  | 582600ATE | | aspirin | |
|  |  | 667500ACE | | aspirin | |
|  |  | 110801ATB | | aspirin (encapsulated) | |
|  |  | 110802ATB | | aspirin (encapsulated) | |
|  |  | D87600ATE | | aspirin (encapsulated) | |
|  |  | 110901BIJ | | aspirin lysine | |
|  |  | 110902BIJ | | aspirin lysine | |
|  |  | B21200ATB | | aluminum aspirin | |
|  |  | 259000ATB | | aluminum aspirin | |
|  |  | 259100ACH | | aluminum aspirin | |
|  |  | A20100ATB | | aspirin encapsulated | |
| Steroids | | 116401ATB | | betamethasone 500㎍ | |
|  |  | 116502BIJ | | betamethasonesodiumphosphate 4mg | |
|  |  | 296900ATB | | betamethasone 250㎍ | |
|  |  | 140801ATB | | deflazacort 6mg | |
|  |  | 141901ATB | | dexamethasone 500㎍ | |
|  |  | 141903ATB | | dexamethasone 750㎍ | |
|  |  | 141904ATB | | dexamethasone 4mg | |
|  |  | 142201BIJ | | dexamethasonesodiumphosphate 5mg | |
|  |  | 142202BIJ | | dexamethasonesodiumphosphate 4.37mg | |
|  |  | 160201ATB | | fludrocortisoneacetate 100㎍ | |
|  |  | 170901ATB | | hydrocortisone 10mg | |
|  |  | 170906ATB | | hydrocortisone 5mg | |
|  |  | 171201BIJ | | hydrocortisonesodiumsuccinate 100mg | |
|  |  | 171202BIJ | | hydrocortisonesodiumsuccinate 250mg | |
|  |  | 193302ATB | | methylprednisolone 4mg | |
|  |  | 193305ATB | | methylprednisolone 1mg | |
|  |  | 193501BIJ | | methylprednisoloneacetate 200mg | |
|  |  | 193502BIJ | | methylprednisoloneacetate 40mg | |
|  |  | 193601BIJ | | methylprednisolonesodiumsuccinate 125mg | |
|  |  | 193602BIJ | | methylprednisolonesodiumsuccinate 250mg | |
|  |  | 193603BIJ | | methylprednisolonesodiumsuccinate 40mg | |
|  |  | 193604BIJ | | methylprednisolonesodiumsuccinate 500mg | |
|  |  | 217001ATB | | prednisolone 5mg | |
|  |  | 217003ASY | | prednisolone 1mg | |
|  |  | 217004ASY | | prednisolone 3mg | |
|  |  | 217302BIJ | | prednisolonesodiumsuccinate 250mg | |
